# Engaging rural Australian communities in National Science Week helps increase visibility for women researchers

**DOI:** 10.1098/rsos.170548

**Published:** 2017-10-18

**Authors:** Margaret C. Hardy, Mathilde R. Desselle, the 2016 Catch a Rising Star Consortium

**Affiliations:** 1 Division of Chemistry and Structural Biology, Institute for Molecular Bioscience, The University of Queensland, Brisbane, Queensland, Australia; 2 Centre for Superbug Solutions, Institute for Molecular Bioscience, The University of Queensland, Brisbane, Queensland, Australia; 3 Jennifer Allen, Cetacean Ecology and Acoustics Laboratory, The University of Queensland, Brisbane, Australia; Katherine T. Andrews, Griffith Institute for Drug Discovery, Griffith University, Nathan, Queensland, Australia; Dani J. Barrington, School of Public Health, The University of Queensland, Brisbane, Australia (current address: School of Civil Engineering, University of Leeds, Leeds, UK); Danielle Borg, Inflammatory Disease Biology and Therapeutics, Mater Research Institute, The University of Queensland, Translational Research Institute, Woollongabba, Brisbane, Australia; Kaylene Butler, School of Earth and Environmental Sciences, The University of Queensland, Brisbane, Australia; Rebecca Colvin, School of Geography, Planning and Environmental Management, The University of Queensland, Brisbane, Australia; Tarni Louisa Cooper, School of Veterinary Science, The University of Queensland, Gatton, Australia; Emily Furlong, Division of Chemistry and Structural Biology, Institute for Molecular Bioscience, The University of Queensland, Brisbane, Australia; Honor Hugo, Institute of Health and Biomedical Innovation, Queensland University of Technology, Translational Research Institute, Woollongabba, Brisbane, Australia; Elecia Johnston, Molecular & Cell Biology, College of Public Health, Medical & Veterinary Sciences, James Cook University, Townsville, Australia; Gwenllian Iacona, Centre of Excellence for Environmental Decisions, The University of Queensland, Brisbane, Australia; Carly Kenkel, Australian Institute of Marine Science, Townsville, Queensland, Australia (current address: Department of Biological Sciences, University of Southern California, Los Angeles, CA, USA); Caitlin Kuempel, Centre of Excellence for Environmental Decisions, School of Biological Sciences, The University of Queensland, Brisbane, Australia; Amie Khosla, School of Mathematics and Physics, The University of Queensland, Brisbane, Australia; Danette Langbecker, Centre for Online Health, The University of Queensland, Brisbane, Australia; Jacki Liddle, Queensland Brain Institute and School of Information Technology and Electrical Engineering, The University of Queensland, Brisbane, Australia; Diana Lucia, School of Biomedical Sciences, The University of Queensland, Brisbane, Australia; Vanessa Lussini, School of Chemistry, Physics and Mechanical Engineering, Science and Engineering Faculty, Queensland University of Technology, Brisbane, Australia; Lynn Nazareth, Griffith Institute for Drug Discovery, Griffith University, Nathan, Queensland, Australia; Alison Peel, Environmental Futures Research Institute, Griffith University, Nathan, Queensland, Australia; Megan Saunders, School of Earth and Environmental Science, The University of Queensland Centre for Biodiversity and Conservation Science, Brisbane, Australia (current address: School of Chemical Engineering, The University of Queensland Centre for Biodiversity and Conservation Science, Brisbane, Australia); Meaghan Smith, Faculty of Science and Engineering, University of the Sunshine Coast, Queensland, Australia (current address: GeneCology Research Centre, Faculty of Science and Engineering, University of the Sunshine Coast, Queensland, Australia); Johana Tello Velasquez, Griffith Institute for Drug Discovery, Griffith University, Nathan, Queensland, Australia

**Keywords:** altmetrics, career advancement, career development, equity, research, social media

## Abstract

During a week-long celebration of science, run under the federally supported National Science Week umbrella, the Catch a Rising Star: women in Queensland research (CaRS) programme flew scientists who identify as women to nine regional and remote communities in the Australian State of Queensland. The aim of the project was twofold: first, to bring science to remote and regional communities in a large, economically diverse state; and second, to determine whether media and public engagement provides career advancement opportunities for women scientists. This paper focuses on the latter goal. The data show: (i) a substantial majority (greater than 80%) of researchers thought the training and experience provided by the programme would help develop her career as a research scientist in the future, (ii) the majority (65%) thought the programme would help relate her research to end users, industry partners or stakeholders in the future, and (iii) analytics can help create a compelling narrative around engagement metrics and help to quantify influence. During the week-long project, scientists reached 600 000 impressions on one social media platform (Twitter) using a program hashtag. The breadth and depth of the project outcomes indicate funding bodies and employers could use similar data as an informative source of metrics to support hiring and promotion decisions. Although this project focused on researchers who identify as women, the lessons learned are applicable to researchers representing a diverse range of backgrounds. Future surveys will help determine whether the CaRS programme provided long-term career advantages to participating scientists and communities.

## 1. Introduction

According to recent estimates, 75% of the fastest growing occupations in Australia require science, technology, engineering and mathematics (STEM) skills [1]. In Australia, the emphasis on ‘engagement’ and ‘impact’ in policy has moved beyond buzzwords, with the 2016 National Innovation and Science Agenda (http://www.innovation.gov.au/) dedicating AU$1.1 billion over 4 years to help Australia embrace technological change.

In 2011, 2.3 million individuals in Australia were employed in the STEM workforce: 32% of those were university qualified and 68% vocational education and training qualified [2]. For comparison, 5.7 million individuals in Australia had non-STEM qualifications. Indigenous students enrolled in higher education degrees in STEM represent less than 1% of enrollments and approximately 1% of higher education enrollments overall [3].

In Australia, distance is an integral concept to policy as the distances individuals are required to travel to access services, including education, can be considerable. Queensland is the second largest state in Australia, at approximately 2 million km^2^ (approx. 2.7 times the size of Texas, or seven times the size of Great Britain). Indigenous persons are approximately 3% of the Australian population, and approximately 28% of the Australian Indigenous population lives in Queensland.

The Australian Government has created a classification system that incorporates the values from the Accessibility/Remoteness Index of Australia (ARIA+), with 1 km grid data from census districts, to determine whether an area is part of a major city, regional Australia or remote Australia. Values within the classification system range from 0 (high accessibility) to 15 (high remoteness), and are based on road distance measurements to the nearest Australian Department of Health Service Centre. ARIA+ is a geographical indicator of remoteness and excludes any confounding socio-economic and population-based factors and is consistent over time.

Queensland is a large, decentralized state, and the levels of access to and outcomes for education vary considerably between regions. Educational outcomes and attainment are positively correlated with remoteness: for example, students of Queensland government schools living in major cities were less likely to score below the national minimum standard for all three domains (reading, numeracy and writing) than students living in areas classified as very remote [4]. For a specific example, in 2011 9% of Queensland students scored below the national minimum standard for reading statewide, compared with less than 2.5% in the inner western suburbs of the state capital, Brisbane. In contrast with this, 25% of students in Far North Queensland and 19% in the Northern Outbackscored below the national minimum standard for reading [4]. The same data report Aboriginal and Torres Strait Islander students living in very remote areas of Queensland were more than twice as likely to score below the national minimum standard across the three domains of numeracy, reading and writing compared to Aboriginal and Torres Strait Islander students in major cities (34% in very remote areas compared with 14% in major cities). Restricted access to education and other services in remote areas, therefore, disproportionately affects Indigenous Australians.

In 2014–2015, the number of students from regional and remote areas of Australia commencing higher education decreased by 1.6% and 4.0%, respectively; during the same time, the number of low socio-economic status students commencing increased by 0.1% [5]. Indigenous persons are 27 times more likely to live in very remote areas of Australia than non-Indigenous persons (13.7 compared with 0.5%), while non-Indigenous Australians are 2.4 times as likely to live in major city areas than Indigenous Australians (71.3 compared with 34.8%) [6].

Distinct from formal educational instruction, National Science Week in Australia (www.scienceweek.net.au) is a week-long public campaign to celebrate science and technology: ‘It provides an opportunity to acknowledge the contributions of Australian scientists to the world of knowledge. It also aims to encourage an interest in science pursuits among the general public, and to encourage younger people to become fascinated by the world we live in' (https://www.scienceweek.net.au/faq/#q1). Held every August since 1997, the programme is supported by the Australian Government and a number of national partners. In 2016, more than 2000 activities took place with more than 1 million participants in science events across the country. The goal of National Science Week is to reach the general public, not just science enthusiasts already engaged with STEM. As part of this effort, the National Science Week programme works with a range of traditional media outlets to highlight Australian science and innovation in August.

Historically, a scientist's interaction with the media was thought to be a matter of personal preference. However, for modern scientists public engagement is increasingly considered an integral part of a researcher's role and a specific hallmark of leadership [7]. In a 2014 survey of American scientists, 43% of respondents thought it was important or very important for scientists to get coverage of their work in traditional TV and print news media; 22% thought it was important or very important for career advancement in their discipline to promote their findings on social media [8]. Social media not only includes platforms like Twitter, Facebook and Instagram, but also blogging through both branded (e.g. for a journal or professional society) and personal outlets. Further analysis of the survey data illustrated that the likelihood of engaging with the non-scientist public was strongly correlated with a belief that media attention was important for career advancement [9].

Metrics can also be influenced by working with the media, most notably through altmetric and other measures that incorporate mainstream news, research blogs, Wikipedia entries and mentions on social networks. Public dissemination of scientific results increases with seniority, according to previous work, and scientists with a higher *h*-index are slightly more active in industry collaborations [10]. Discussing new discoveries on social media and talking with online media outlets have been shown to increase a researcher's *h*-index [11]. Although the apparent utility of the *h*-index as a measure of career success varies between research disciplines [12,13], it is one example of a metric commonly used for hiring and promotion that can be influenced by public engagement. A substantial majority (72%) of medicine and paediatric department chairs favoured meeting the ‘educational scholarship’ requirement for the promotion and tenure process through involvement in a journal-based blog [14].

The debate over whether media attention independently increases the impact of a research paper (the publicity hypothesis), or whether the media merely ‘earmarks’ outstanding research early on (the earmark hypothesis), has been examined in depth and the publicity hypothesis was found to be supported [15].

The impact of being seen in the media has particular implications for women scientists, in negotiating the ‘pushy or princess’ stereotype when appearing publicly as an expert [16]. However, we propose that it is not her ability to serve as an expert, but the public's acceptance of her credibility as an expert, that presents the challenge.

The importance of providing directed training that is focused on community engagement, not just media training, for Australian postdoctoral researchers has been outlined recently [17]. Data collected in the United Kingdom show 53% of people graduating with a PhD in a STEM field move immediately into a career outside science; less than 0.5% of STEM PhD graduates earn the rank of ‘professor’ [18]. In New Zealand, 75% of STEM graduates move immediately into a career outside of science post-PhD; fewer than 2% of STEM PhD graduates ultimately become a professor [19]. Career advancement for women scientists is particularly fraught with difficulty, and in Australia 30% of university staff above Senior Lecturer (Level C) were women [20]. In an environment where becoming a professor is the exception, rather than the norm, training programmes for PhD students and early career researchers should teach the skills required to explain scientific concepts clearly and concisely to the public.

Although the positive impact of one-off science engagement events on regional Australian communities has been previously demonstrated [21,22], one question that has been largely unexplored in the literature is whether participating in such events is beneficial for advancing the career of the participating scientists. By hosting in-class and in-community panel discussions, the Catch a Rising Star (CaRS) programme framed the teaching and learning of science both *as* inquiry and *by* inquiry [23], which represents a departure from traditional science fair or science circus events. Previous research has established that stereotypes of scientists as ageing white men who are ‘pale, male and stale’ have a negative impact on women undergraduate students in computer science [24]. When asked to draw a scientist or an engineer, a cohort of middle schoolers in the United States drew women as scientists 40% and as engineers 50% of the time [25]. This is a vast improvement on the original experiment from 1960s to 1970s in Canada, in which primary school students drew scientists as women only 0.5% of the time, and all those drawing scientists as women were girls [26].

### 1.1. The Catch a Rising Star programme

The CaRS 2016 programme had two goals: to provide a more diverse model of Australian STEMM (science, technology, engineering, mathematics and medicine) by sending women researchers into regional and remote communities, and to determine whether public engagement and media work could contribute to career development for the research scientists. In-community, researchers not only talked about their individual fields specifically, but also about science and technology more generally. In agricultural regions, scientists talked about how technology has improved sustainability and productivity; in mining communities, participants explored the idea of a circular economy and how to conserve biodiversity while creating jobs.

## 2. Methods

### 2.1. Background on participants

Of the 46 researchers who originally applied over the four-week period expressions of interest were open, 16 withdrew before the 2-day mandatory training workshop. Of the 30 researchers who attended the workshops, 28 participated in the site visits. Of the 28 who participated in site visits during National Science Week, 23 (50% of the applicants) are co-authors on this paper.

### 2.2. Background on the project

The project was supported by a single grant for National Science Week awarded to the Chief Investigators M.C.H. and M.R.D. in 2016. The project was managed by two fixed-term contract employees (M.C.H. and M.R.D.). M.C.H. is a research-focused academic, and M.R.D. is a programme manager focused on marketing and outreach. Travel was coordinated through a travel agent from Campus Travel at The University of Queensland.

### 2.3. Training workshop

The training workshop ran over a day and a half. The first event was a 2 h media panel, titled *Queensland Scientists Meet the Media*; this panel was open to the public. We are grateful to Kathy McLeish (ABC News, for radio, television and online programmes), Rhianna Patrick (ABC Local Radio), Bernadette Young (612 ABC Brisbane Radio) and Michael Lund (*The Conversation*) for volunteering their time. Local and national media experts volunteered their time to talk about what makes a good science story, how they find their science stories and provided practical strategies to help scientists communicate their findings to the public.

The second day of the training began with two morning workshops. The first was led by M.C.H. and focused on measuring altmetrics for career progression and how engagement with the media and, by extension, the broader community can build meaningful relationships with industry partners, the public and other stakeholders. The goal of this session was to provide a framework to speak with the public (either on social media or through traditional media outlets) with an objective of obtaining grant or other funding, with a particular focus on how to build meaningful collaborations with other scientists through increasing one's visibility online. The second workshop was led by M.R.D. and focused on public speaking skills for community events. The goal of this session was to practice voice and posture exercises for impactful presentations to help manage nervousness. By developing a personal and engaging style, each participant could organize her thoughts for specific programme audiences, including students, the media and the non-scientist public.

The second half of the day was spent on introducing the teams that had been put together by M.C.H. and M.R.D., based on dates of availability and the inclusion of a range of scientific disciplines within each site visit. In this part of the workshop, teams were tasked with picking a 2-day period they wanted to travel (in some areas, taking into account the limited flight service schedules) and mapping out potential hosts for their community visit, including schools, libraries and community groups. Scientists were initially allocated into groups of four, with a goal of accommodating dates available and representing a broad range of disciplines at each of the nine sites. At the training workshop people reorganized their groups as needed. Once groups were formed, they were self-led in building relationships with the local hosts and planning the in-community visit.

After the workshop, participants were asked to write a ‘Letter to your Teenage Self’, and all the letters were made into a single document (PDF available on Figshare, https://doi.org/10.6084/m9.figshare.4867103) that was posted online for the communities to use to get to know the researchers. Participants were also asked to draft an experiment from their area of expertise that could be done at home or at school, with items that could be easily found in a supermarket or hardware store.

### 2.4. Community visits

In order to make meaningful connections between the researchers and the communities, a community-led approach was implemented. An online expression of interest form was emailed to local schools, community groups and libraries. Our focus was on non-denominational public sector or non-profit groups. We made particular efforts to visit schools with majority enrollments of Aboriginal and Torres Strait Islander students. As Internet access is unreliable in many areas, follow-up telephone calls and Facebook messages proved an effective way to proceed in many instances. Where possible, we arranged our visit around existing programming (e.g. trivia nights at the local public house or ‘pub’ or similar). Most of the sites had at least two events, one for the public and one for one of the local schools (mostly secondary schools, but also some primary schools).

### 2.5. Logistics

The National Science Week website was used to advertise events (searchable by event type or location), and also provided templates for certificates and flyers. Individual community groups also publicized events through their usual channels. The Institute for Molecular Bioscience at The University of Queensland administered the grant, with M.C.H. and M.R.D. as programme managers. A commercial travel agent based at the University was used to arrange flights and accommodation. Because scientists were travelling as professionals, PhD students and staff members confirmed their personal and public liability insurance with their respective employers in advance of their trip. In Australia, Medicare provides emergency medical coverage to citizens and permanent residents, and for international students and visa-holders private healthcare including emergency cover is a visa requirement. Many communities were chosen because of their remote location, and in case of a medical emergency (both travel-related accidents and other types of incident, like snakebite) appropriate coverage would be critical.

### 2.6. Survey

The survey questions were drafted with input from all the authors. The survey questions and responses are available in the electronic supplementary material data (available on Figshare, https://doi.org/10.6084/m9.figshare.4867103). Programme participants, but not the programme co-organizers M.C.H. and M.R.D., took the self-assessment focused survey; M.C.H. anonymized and analysed the data.

## 3. Results

**Figure 1.**
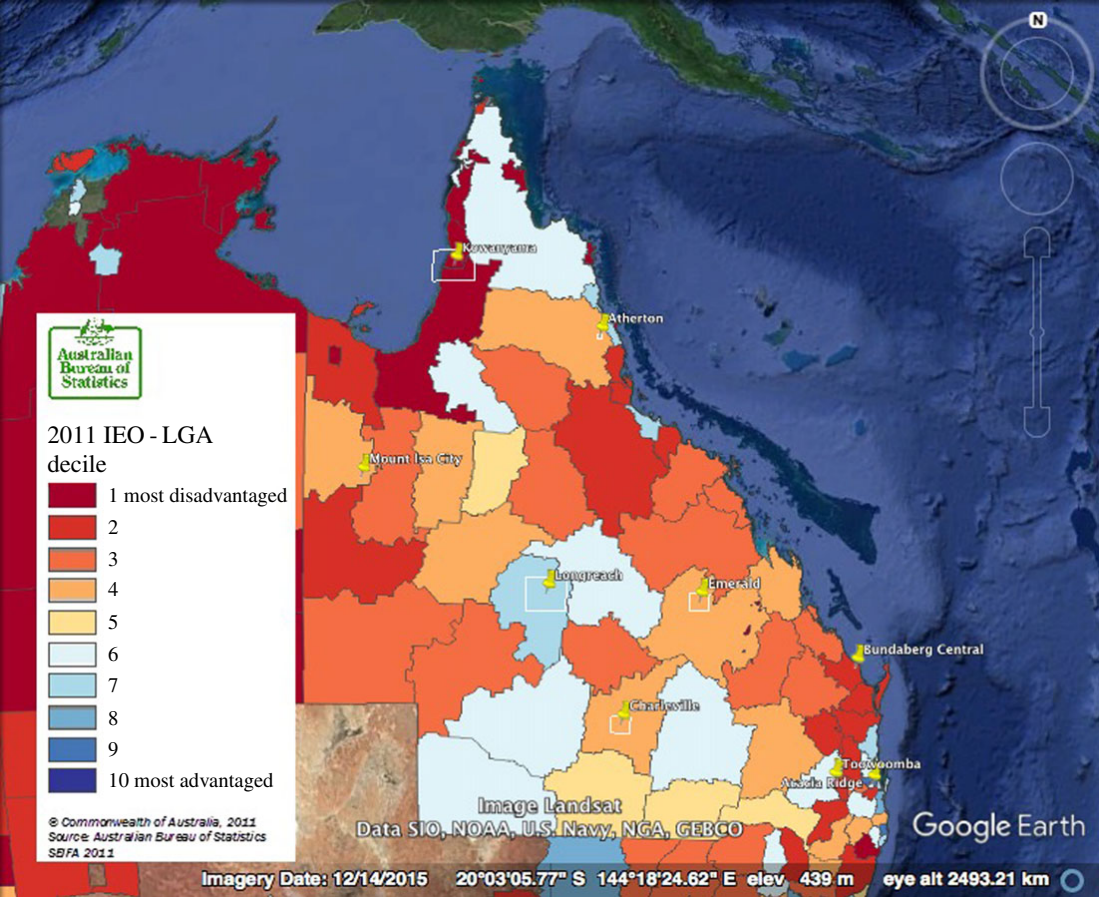
A map of the Australian State of Queensland, with the most recent available Index of Education and Occupation (IEO) overlaid and mapped by local government area (LGA).

Survey respondents represent those researchers who received targeted science communication training and visited each of the nine community sites: Atherton and surrounding towns (three respondents), Brisbane suburbs (two), Bundaberg (four), Charleville (three), Emerald (three), Kowanyama (three), Longreach (one), Mt Isa (two) and Toowoomba (two). As programme managers, M.C.H. and M.R.D. did not participate in the survey. An overview of the communities visited as part of the project illustrates the diversity of socio-economic circumstances in regional and remote communities (table 1, figure 1).

**Table 1.** An overview of the socio-economic and remoteness indicators for the communities visited. The Australian Bureau of Statistics Socio-Economic Indexes for Areas, which draws data from the 2011 Census, allows regional patterns of socio-economic stratification to be determined. The census divides the country into 577 local government areas, and each of those are ranked by percentile; for the following determination the Index of Education and Occupation was used, which incorporates characteristics including the proportion of people with a higher education or those employed in a skilled occupation. The remoteness classification is based on the Australian Standard Geographical Classification.

map location	community	percentile	remoteness classification
1	Atherton and surrounds	36	outer regional
2	Brisbane suburbs	20	major city
3	Bundaberg	13	inner regional
4	Charleville	37	remote
5	Emerald	36	outer regional
6	Kowanyama	8	very remote
7	Longreach	69	very remote
8	Mt Isa	37	remote
9	Toowoomba	60	inner regional

**Figure 2.**
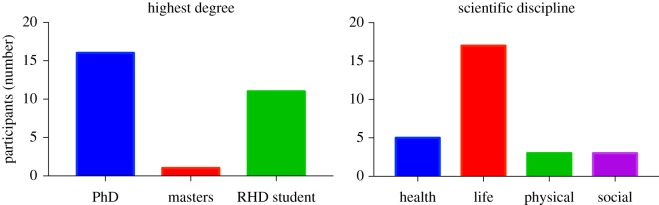
An overview of the demographics of participating researchers. An RHD student is a research higher degree student, enrolled in either a masters or PhD programme. Health Sciences include medicine/dentistry, nursing/health professions, pharmacology/toxicology, veterinary science/medicine; Life Sciences include agricultural and biological sciences, biochemistry, genetics, molecular biology, environmental science, immunology/microbiology, neuroscience; Physical Sciences & Engineering include chemistry, computer sciences, Earth/planetary sciences, engineering, materials science, mathematics, physics; and Social Sciences include business, accounting, economics, psychology, social sciences.

The researchers represent a diverse range of scientific backgrounds (figure 2). More than 55% of the participants hold a PhD, and nearly 40% were research higher degree (RHD) students. The majority of respondents come from the life sciences (60%), with the remainder from health (18%) and the physical and social sciences (11% each). All of the researchers were from non-profit entities, either universities or government agencies.

On each community visit, researchers planned an average of three events: generally, one community visit, one school visit including hands-on experiments and one ‘science at the pub’ type event. Both adults (aged over 18) and young people (aged less than 18) were reached by the programme in person in approximately equal proportions; an estimated total of 5250 people attended events in person. According to the Queensland Government the most recent population estimate for the state was 4.8 million [27], so our project with 28 scientists reached approximately 0.1% of all Queenslanders in person. Unlike other programmes, our programme disproportionately reached people who are traditionally underrepresented in higher education and careers in STEMM, including Indigenous persons, persons from disadvantaged socio-economic areas, and persons from remote and regional areas.

The use of social media proved particularly successful for the programme. Over the 48 h period when researchers were in each community, programme participants focused social media efforts on Twitter to promote that particular visit. Over the 6 days that researchers were visiting communities we also used Twitter to post about National Science Week and higher education and careers in STEMM more generally. This enabled us to focus our efforts centrally and to interact with communities, the media and each other even while we were geographically separated. Using free analytics software (Tweetreach, https://tweetreach.com/) and a dedicated hashtag (#QLDStars), we were able to track the number of impressions and accounts reached during the week. On average, every 2 days we reached 40 000–60 000 individual accounts and had an exposure of 185 000–215 000 impressions, for a total of approximately 600 000 impressions on Twitter over the course of National Science Week. We achieved this considerable reach although most of the participating researchers each had between 200 and 1000 Twitter followers.

The traditional media reach for the programme included at least 11 interviews with local and regional radio stations and newspapers, resulting in a total audience of more than 127 000 people (data provided by The University of Queensland). In addition, M.C.H. was part of a larger national media campaign organized by the National Science Week programme staff through a production house specializing in non-profit work, and she was the featured scientist in 12 of the 33 interviews that made up the campaign. During these interviews, M.C.H. discussed her own research but primarily talked about the CaRS National Science Week programme. Overall, the 33 radio interviews were broadcast 572 times in 282 unique markets; the 12 interviews by M.C.H. generated 253 broadcasts reaching every state and territory in the country (data provided by MediaHeads). Accordingly, the interviews featuring M.C.H. were 44% of the total broadcasts for the national publicity platform, and reached 90% of the broadcast markets. One of those interviews was on the national radio station Triple J during the weekly *Science Hour* programme. Because Triple J is a government funded national station it is difficult to estimate the audience size, but in the most recent annual report the average weekly audience for *Science Hour* (aged 10+) was 1.89 million [28].

Overall, a substantial majority (greater than 85%) of researchers thought participating in the programme was somewhat or very useful for their career development. More than 80% of researchers also thought the training and experience provided by the programme would help develop her career as a research scientist in the future. A majority (65%) of researchers thought the training and experience the programme provided would help relate her research to end users, industry partners or stakeholders in the future. One participant (M. Smith) reported that a national free-to-air children's science television programme (Scope) made contact with her to film a dedicated segment on her research after hearing about her work on Twitter.

Before the CaRS programme, approximately 70% of participants had little or no experience working with the media. During the programme, approximately 45% of the participants worked with traditional media outlets (print, radio, TV or online news). More than 80% of participants thought the training and experience provided by the programme helped them better relate their research to the public and to students.

The number of participants who use social media for academic or professional purposes (e.g. finding collaborators, publicizing novel results) increased by 32.6% after the programme, and 100% of participants reported that the use of social media during the programme raised (87%) or somewhat raised (13%) their professional profile. An increase in professional profile was considered an increase in the total number of followers and/or engagements on social media following a post about a researcher's findings or a topical post in her area of research.

Overall, 87% of participants somewhat or strongly agree their expectations of the programme were met, and the remainder were neutral. All of the researchers would be interested in participating in the programme again, if it were to run again in the future.

## 4. Discussion

The project results demonstrate that a directed training programme can help scientists develop the technical proficiency to communicate effectively with the public and with the media. Most notably, a substantial majority (greater than 85%) thought the programme was somewhat or very useful for their career development, and more than 80% thought the training and experience provided would help develop her career as a research scientist in the future. A majority (65%) of researchers thought the training and experience the programme provided would help relate her research to end users, industry partners or stakeholders in the future. These linkages are not only critical for future grant success as academic researchers, but also help showcase skills needed to collaborate in government and industry roles. The data illustrate that dedicated training programmes like this one can help reverse the historical tradition of minimizing the research contributions of women scientists, a practice with a considerable historical legacy in Australia and New Zealand [29].

Overall, the programme illustrates the power of curating an event under a single hashtag, and also provides useful metrics for reporting to funding bodies and for use in future grant applications. Additionally, the national reach of the programme illustrates the benefits of working with production houses and media professionals to bring science to the public. In the 12 months since the 2016 National Science Week programme ended, programme participants have reported a number of professional promotions and the propagation of a number of public engagements with science grant funded projects. Future work can continue to examine whether the credibility of participating researchers was increased long term, and whether participating scientists advanced their careers as a result of the skills gained through their participation in the programme.

The programme has continued in 2017, with another cohort of 18 women scientists affiliated with Queensland research institutions visiting six regional communities (Bundaberg, Cairns and Atherton, Kowanyama, Mackay and Moranbah, Mount Isa and Julia Creek, Townsville, and Magnetic Island). A dedicated Twitter account (@ScienceStarsAU) and email account (ScienceStarsAU@gmail.com) were created to centralize communications for the team. Lists of members in the 2016 (https://twitter.com/sciencestarsau/lists/qld-stem-stars-2016) and 2017 (https://twitter.com/sciencestarsau/lists/qld-stem-stars-2017) cohorts are available online. Storify, a free online platform for content aggregation, was used to create a digital narrative for the 2016 (https://storify.com/DrMaggieHardy/qldstars-storify) and 2017 (https://storify.com/sciencestarsau/2017-catch-a-rising-star-in-queensland) programmes.

If engagement really is a key priority for research, as many government agencies and funding bodies suggest, this type of project should be recognized as such. Note the project was not considered part of the Higher Education Research Data Collection (HERDC) research income data tracked and submitted by the university. Despite being a sizeable and competitive grant, because the project was considered non-HERDC it did not count towards the research track record of the chief investigators. Further, the funding guidelines prohibited a stipend for administrative staff, the organizers or participating researchers; future funding could permit hiring an administrative assistant (even part time) for the duration of the project to manage details like scheduling, travel and accommodation arrangements. As these tasks are not what researchers are paid to do, these tasks were not even official ‘service’ tasks and were truly voluntary.

There is still much to be learned about the most effective way to engage underserved communities with science and traditionally underrepresented groups with higher education and careers in STEMM. This project begins the conversation by demonstrating that training and outreach programmes, when paired with media attention and a strategic social media campaign, can increase visibility for research scientists from a diverse range of disciplines. This paper demonstrates the positive impact a short-term, focused engagement initiative has on visibility for women scientists, and lays the groundwork for funding bodies to support training programmes that will help prepare research scientists to engage in an inclusive way with constituent communities.

## Data accessibility

Our data are available in Figshare: https://doi.org/10.6084/m9.figshare.4867103. For questions or additional information contact the programme coordinators via email (ScienceStarsAU@gmail.com) or on Twitter (@ScienceStarsAU).
